# Toxic effects of detected pyrethroid pesticides on honeybee (*Apis mellifera ligustica* Spin and *Apis cerana cerana* Fabricius)

**DOI:** 10.1038/s41598-022-20925-x

**Published:** 2022-10-06

**Authors:** Qiongqiong Liu, Qibao He, Shiyu Zhang, Yuhao Chai, Quan Gao, Jinjing Xiao, Qingkui Fang, Linsheng Yu, Haiqun Cao

**Affiliations:** 1 School of Plant Protection, Anhui Agricultural University, 130 West Changjiang Road, Hefei, 230036 Anhui People’s Republic of China; 2 Anhui Province Key Laboratory of Crop Integrated Pest Management, Hefei, 230036 China

**Keywords:** Environmental sciences, Risk factors, Chemistry

## Abstract

To obtain the presence of environmental contaminants in honeybee and compare the toxicity of the detected pesticides to *Apis mellifera ligustica* Spin and *Apis cerana cerana* Fabricius. In this work, 214 honeybee samples were collected to simultaneous monitoring 66 pesticides between 2016 and 2017 in China. A modified QuEChERS extraction method coupled with multi-residue analytical methods by Ultra-high-performance liquid chromatography-tandem mass spectrometry (UPLC-MS/MS) and Gas chromatography-mass spectrum (GC–MS). Among, four pyrethroid pesticides were selected to test and compare the acute oral toxicities of two honeybees. And the survival risk of beta-cypermethrin was analyzed to them. Using this method, 21 compounds were detected, including 3 neonicotinoids, 5 pyrethroids, 5 organophosphorus and 8 others. Importantly, detected frequencies of pyrethroid pesticides were accounted for 53.3%. Among, acute toxicity values (LD_50_) of four pyrethroid pesticides to the *A.m. ligustica* were higher than of that the *A.c. cerana*. When they were exposed to the same concentration of beta-cypermethrin (0.2906 mg/L), the survival rate of the *A.m. ligustica* (40.0%) was higher than the *A.c. cerana* (18.9%). Our work is valuable to analyze multiple pesticide residues of honeybees and evaluate the survival risk of two honeybee species, which also provides a basis for the risk assessment.

## Introduction

The honeybee pollinating plants (economy crops, fruiters, vegetables, and so on) to serve agriculture and the global ecosystem, while producing bee products (pollen, honey, royal jelly etc.) with high economic value^1,2^. Meanwhile, they have been providing these functions worth approximately $200 billion for farmers of food production^3^. *A.m. ligustica* (western honeybees) and *A.c. cerana* (eastern honeybees) are two main crop pollination populations in China^4^. *A.m. ligustica,* with high reproduction rate and strong honey production ability, was frequently often used to assess the risk of insecticides. *A.c. cerana* was native species which cultured for long times in the mountain areas of south of China, which have relatively little reports to assess the toxicity of insecticides^5^.

The phenomenon of significant honeybee population declines has attracted much scientific and public attention since 2006. And the synergistic action of several factors including new and re-emerging pathogens, nutrition stress, heavy metal, environmental pollutions and besides these factors, the extensive use of pesticides all contribute to these declines^6,7^. Likewise, honeybees are exposed to a wide range of compounds, including pesticides (neonicotinoid insecticides and pyrethroid pesticides) while foraging in the agricultural cropping systems or consuming contaminated food stocked in the hive^8^. Furthermore, pesticides cause chronic adverse effects to honeybees including impairment of physiology function, and disruption of foraging, olfactory, learning and memory performance^9–13^.

To better investigate the effect of pesticides in the decline of the honeybee population, several reports have developed determination methods for the analysis of pesticides in honeybee bodies. More than 14 relevant pesticides were detected in honeybee samples from 0.3 to 81.5 ng/g by LC–ESI–MS/MS^14^. In another report described two methods based on LC–MS and GC–MS that detected 19 compounds from 145 honeybee bodies^15^. The method was developed to determine 11 pesticides by GC–MS, which coumaphos and tau-fluvalinate were the most frequently detected pesticides^16^. Colony losses were attributed to the presence of pesticides of honeybee bodies, as honeybees are proven bio-samplers in their foraging area^17,18^.

These results of what bodies of honeybee matrix detected multiple pesticides provide evidence to explain its role in the decline of honeybees. Consequently, it is important to develop sensitive and reliable methods to detect the pesticide residues of 214 groups of honeybee samples by GC–MS and UPLC-MS/MS in China. Meanwhile, pyrethroid pesticides, which are the most commonly used class of insecticide in agriculture, exposed to two honeybee species have few experiments focused on^19^. For these concerns, this study was to: (i) develop an analytical method for trace analysis of 66 pesticides and their metabolites in honeybees, (ii) display pesticide pollution in the honeybee environment in various regions of China, and (iii) reveal differences in the sensitivity of pyrethroid pesticides on two honeybee species by combining acute and chronic toxicity under the same conditions.

## Materials and methods

### Chemicals

Individual pesticide standards with a purity of ≥ 97.0%, including chlorothalonil, alachlor, butralin, melachlor, lambda-cyhalothrin, terbufos, oxadiazon, bifenthrin, beta-cypermethrin, terbufos-sulfoxide, quizalofop-p-ethyl, fenvalerate, deltamethrin, fenthion, terbufos-sulfone, and boscalid were obtained from ANPEL Laboratory Technologies Inc. (Shanghai, China). Standard stock solutions of the pesticides were prepared in methanol and stored at − 20 °C. The physical and chemical properties of the tested pesticides are summarized in Table S1.

#### Honeybee sample collection

**Figure 1:**
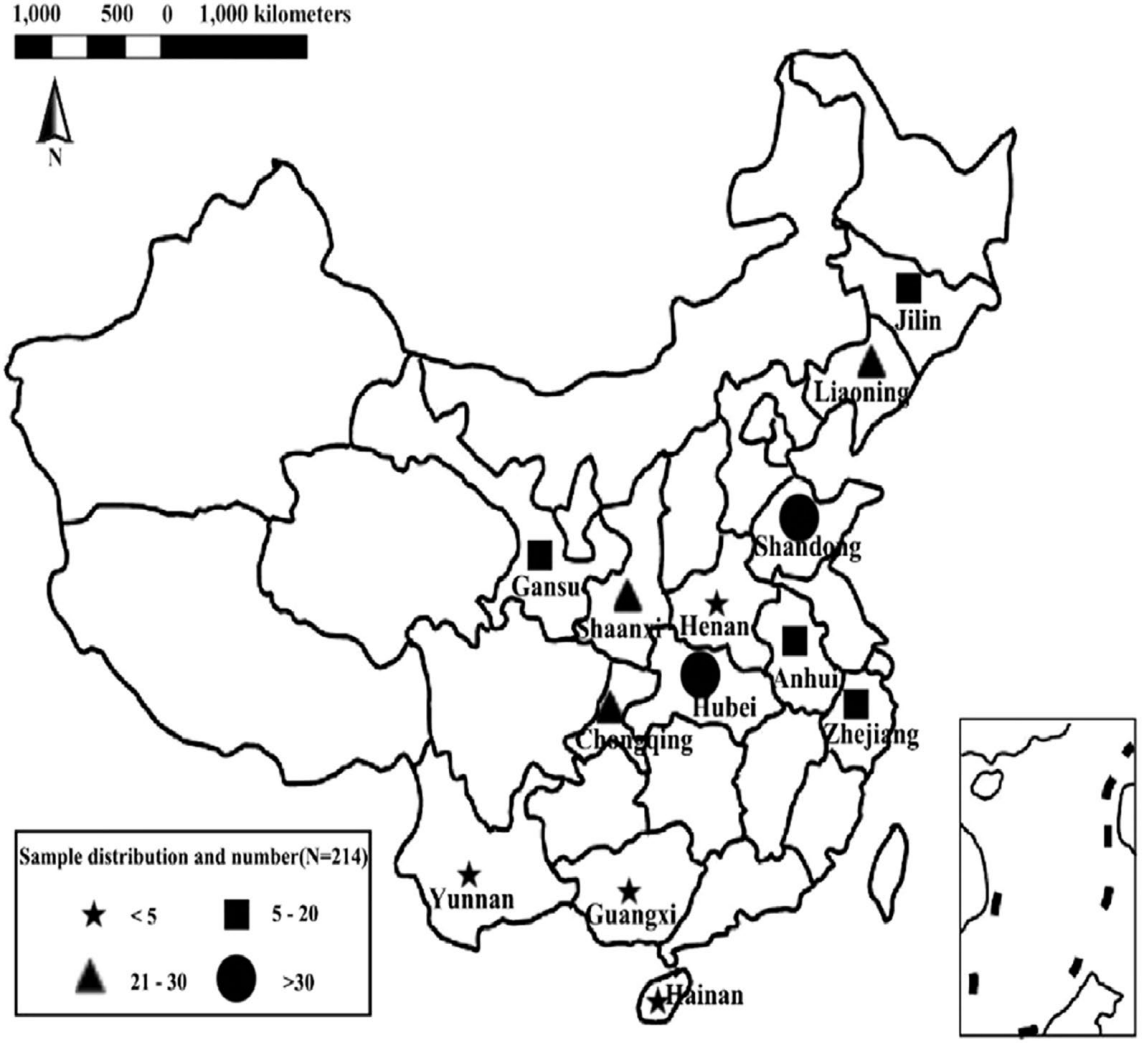
The region of the sample collected in China between 2016 and 2017 (N = 214). Five-pointed Stars represent the number of honeybee samples is less five. Squares represent the number of honeybee samples is between five and twenty. Triangles represent the number of honeybee samples is between twenty-one and thirty. Circles represent the number of honeybee samples is more thirty. We used this URL link (https://cn.bing.com/images/search?q=blank%20map%20china%20and%20provinces&qs=HS&form=QBIRMH&sp=1&pq=blank%20map%20china&sc=6-15&cvid=247BBD3C1C2740E6962B42465D5D5634&first=1&tsc=ImageHoverTitle) and added the fonts and shapes by Adobe Illustrator CC or 2019 Adobe Photoshop CS6.

Total 214 honeybee samples were collected at each experimental apiary of thirteen provinces of China between 2016 and 2017 (Fig. 1). Every apiary randomly collected 50–70 honeybees in 100 mL plastic tube, which were immediately cooled at 0 °C with icepacks (if available) to avoid degradation of active substances. The information of sampling time, location, nectar plants and so on were recorded on the label paper.

#### Samples preparation

Extraction was followed the QuEChERS multiresidue method with a modified version, and then detect 214 honeybee samples which ground in liquid nitrogen^20,21^. In order to detect chemical compounds more widely, it was carried out using GC–MS and UPLC-MS/MS. Pesticide extraction procedures for the GC–MS analysis was shown in Fig. S1. And, sample preparation for the UPLC-MS/MS analysis was depicted briefly: extract 2.0 g honeybee samples with 3 mL water and 10 mL acetonitrile containing 1% acetic acid, shaking vigorously, and add 0.5 g NaCl and 2.0 g MgSO_4_. Centrifuge at 4000×*g* for 5 min and clean up with 1.25 g of the QuEChERS salt kit (PSA: C_18_: MgSO_4_: GCB = (1:1:3:0.15, w/w/w/w)), evaporate 2.5 mL supernatant to dryness and reconstitute in 1 mL acetonitrile.

#### Sample analysis

##### GC–MS analysis

GC–MS was provided by Agilent Technology (USA). A 7890N series gas chromatograph is device which comprise of an Agilent 5977B mass spectrometer selective detector (MSD) and a HP-5 ms capillary column was used to provide the analytical separation. The oven temperature: column temperature began at 60 °C, at 30 °C/min to 180 °C (for 5 min), at 10 °C/min to 250 °C (for 10 min) and then at 10 °C/min to 290 °C (for 10 min). The total run time was 40 min. The carrier gas of gas chromatograph was helium (99.999% purity) with constant float rate of 1.2 mL/min. The injection volume was 1 μL with splitless mode. The injector and transfer line were all run at 290 °C.

Mass detector, source temperature and quadrupole were set at 230 °C and 150 °C, was comprised of electronic impact (EI) mode for performing the MS fragmentation at 70 eV ionization energy. Single ion monitoring (SIM) was used for confirmation and quantitation of the pesticides. Table S2 summarizes three ions (one quantifier and two qualifiers) and retention time monitored for each pesticide.

##### UPLC-MS/MS analysis

UPLC-MS/MS analysis was refereed to Tong's settings conditions. Detection conditions for each compound were displayed in Table S3.

### Pesticide exposure to honeybee

#### Honeybees preparation before experiment

Honeybees (*A.m. ligustica* and *A.c. cerana*) were gained from local apiaries in Anhui agricultural University, in China. Before experiments, the hive was not exposed to any chemical treatments^22^. Honeybee starved in an incubator for approximately 2 h for the food content was equal before experiment^23,24^.

#### Acute oral toxicity assay of four pyrethroid pesticides to honeybees

For the acute oral assay, some doses of each insecticide were prepared after our preliminary experiments. Test solutions of pesticides were prepared by diluting stock solutions with 50% (w/v) sugar solution. And the control was fed with 50% sugar solution with acetone. The amount of treated diet consumed by each group by the difference in weight of the sucrose syrup before and after the experiment. After the acute assay, each group was equipped with a feeder filled with 50% sugar solution without toxicity (27 ± 2 °C and 65 ± 5% RH). The deaths after 48 h treatment were recorded.

#### Survival of the honeybees to beta-cypermethrin

The median concentration (0.0944 mg/L), mean concentration (0.1272 mg/L), and maximum concentration (0.2906 mg/L) of beta-cypermethrin were continuously fed to *A.m. ligustica* and *A.c. cerana* for 10 days. Thirty honeybees were counted into each box (three replicates). The boxes were maintained at 27 ± 2 °C and 65 ± 5% RH, and in darkness. Dead honeybees were recorded daily.

### Data analysis

The linear regression for each compound, the toxicities values (LD_50_) of four pyrethroid pesticides to honeybee, and survival rates of two species of honeybees were analyzed by the software IBM SPSS Statistics 22.0 software (SPSS Inc., Chicago, IL).

The following formula was used to calculate Matrix Effects (ME) (Eq. 1).

1 $$\mathrm{ME}\left(\mathrm{\%}\right)=\left(\left(\frac{Slope \; of \;calibration \; curve \; in \; matrix}{Slope \; of \; calibration \; curve \; in \; solvent} \right)-1 \right) \times 100$$

|ME| < 20% represents mild signal suppression or enhancement effects, 20%  ≤  |M| ≤  50% represents medium effects, and |ME|> 50% represents strong effects^25^.

## Results and discussion

### Method validation

**Figure 2:**
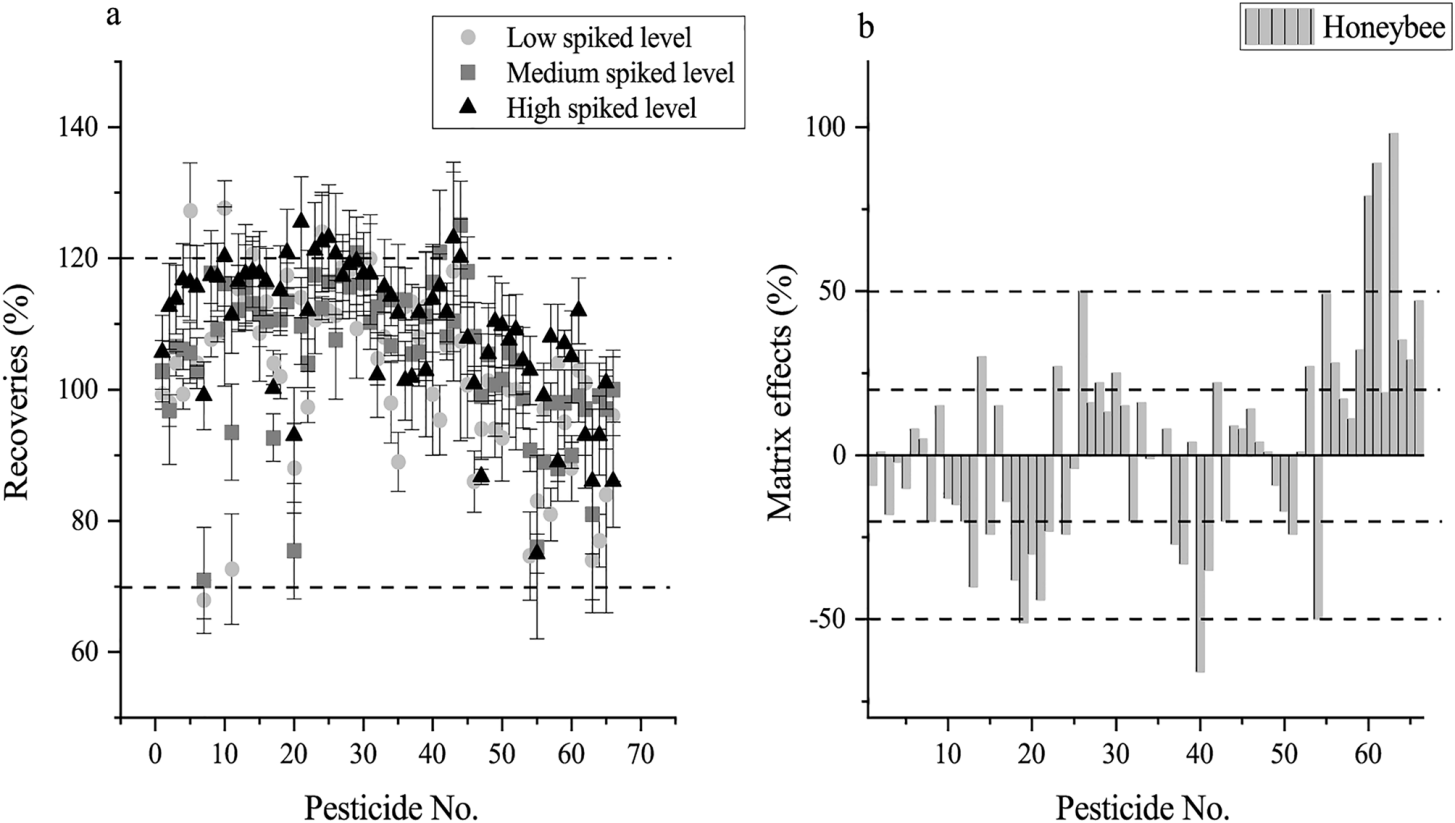
Validation of the method used to investigate 66 pesticides in honeybees. (**a**) Recoveries of 66 pesticides at three different concentration levels for honeybees (n = 5) at low, medium and high levels. (**b**) Matrix effects (%) of the 66 pesticides investigated in the honeybees are shown.

The modified QuEChERS method based on sample extraction and purification is validated. The standard curves for different compounds, obtained with the regression coefficients of more than 0.99 is good linearity results, were established within the concentration range of 2.5–500 ng/g. The limits of detection (LOD) of target compounds were 0.008–2.586 and limits of quantitation (LOQ) were 0.0084–7.758 ng/g (Table S4), and were considered within the concentration achieving a signal-to-noise ratio (S/N) of between 3 and 10. The quantitative method showed excellent performance, which provided mean recoveries within the considered acceptable range 70–120% and relative standard deviations (RSD) below 20% for all targets using matrix-matched calibration curves (Fig. 2a). There were 21 compounds had medium MEs and 9 compounds had high MEs, and 36 pesticides had soft MEs (Fig. 2b and Table S5). Thus, matrix-matched calibration standard solutions were prepared for quantification.

The method should be robust, efficient, and competent to determine pesticide residues in honeybees.A modified QuEChERS extraction method coupled with multi-residue analytical methods by GC–MS and UPLC-MS/MS in this experiment. LC–MS/MS was used for the identification and quantification of the substances in the whole-body residues of the neonicotinoid insecticide imidacloprid in live or dead honeybees^26^. When honeybee matrices are analyzed, the complex nature and the possibility of external interference affecting results should be accounted for^27^.

### Determination of real honeybees samples

**Figure 3:**
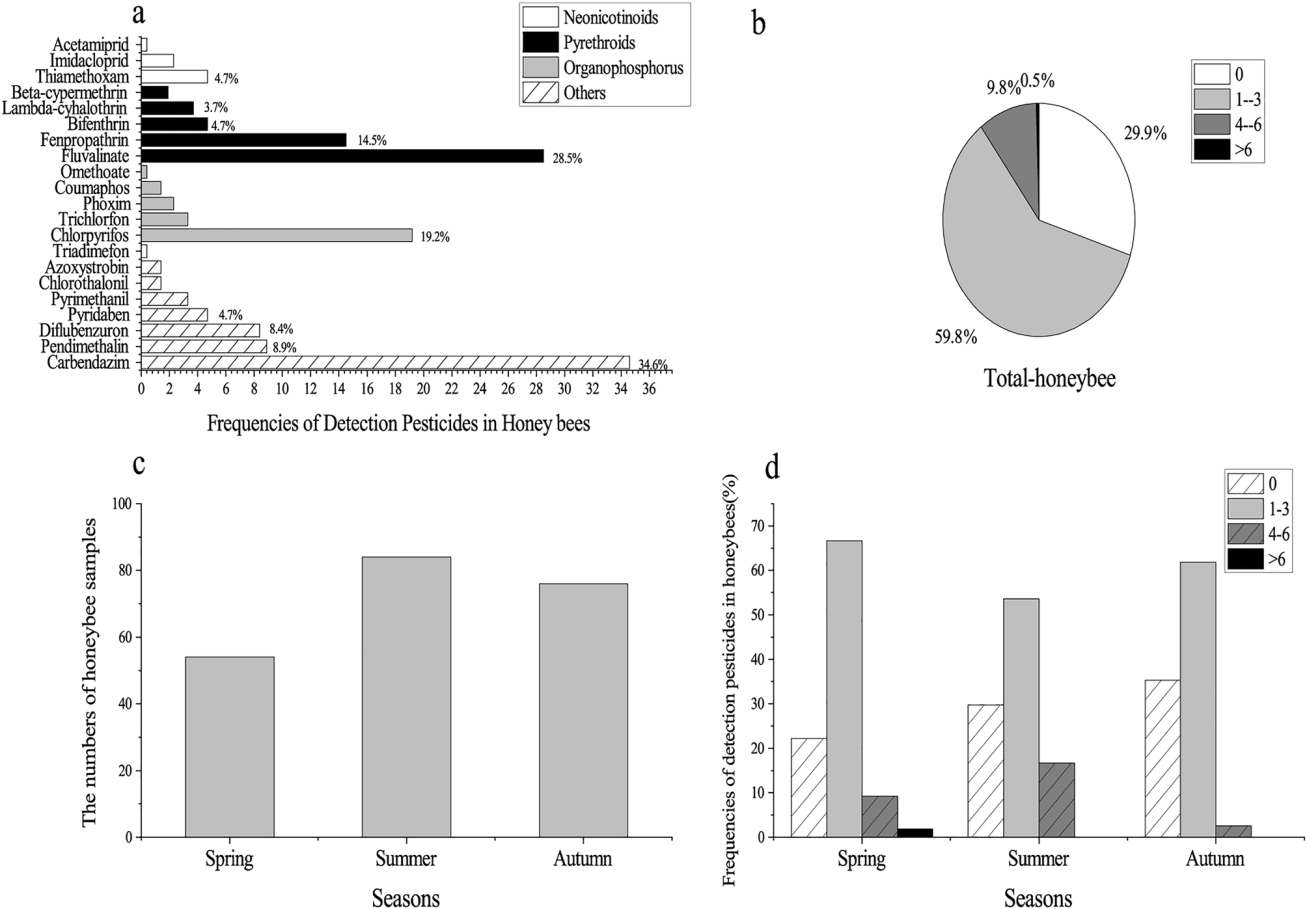
(**a**) Frequencies of detection pesticides in honeybees. (**b**) The species of pesticide residues detected in single honeybee sample. (**c**) The number of samples per season (spring, summer and autumn). (**d**) Distribution of pesticide species detected from honeybee samples in different seasons.

This study was to monitor for 66 common agricultural chemicals and metabolites, as a result of 21 compounds were detected above LOQ in honeybee samples during the 2-year, including 3 neonicotinoids, 5 pyrethroids, 5 organophosphorus and 8 others (Fig. 3a), which detected frequencies of pyrethroid pesticides were accounted for 53.3%. And, there were 10 pesticides with detection rates above 3.7%. It may illustrate that pesticide concentrations of negative samples were mostly less than the LOD and LOQ or none. The most frequently found fungicide was carbendazim (34.6%) (Table 5) and a similar phenomenon was discovered^18^. However, the frequency of detected residues in our study generally disagrees with exposure assessments of collected honeybees reported in the literature, such as chlorpyrifos, phoxim, thiamethoxam, imidacloprid and coumaphos, which may be due to crop planting characteristics and government supervision are different^17,18^. Owning to this, pesticide residues found in honeybees reflect the type of pesticides applied in the agricultural fields and acaricides in the hives. Thiamethoxam and clothianidin were the most frequently found in honeybee samples^28^. Fungicides carbendazim, boscalid, tebuconazole, and so on were in bumble bee bodies^29^. Knowing the effects of simultaneous exposure of honeybees to various pesticides remains a challenge.

Some of 214 honeybees were detected one to six different pesticides or even more than six (Fig. 3b). The highest frequency of detection (59.81%) corresponded to the presence of one to three pesticides. Samples polluted with four to six active ingredients had the frequencies of 9.81%. In addition, more than six pesticides were detected in 0.47% of the samples. Sixty-four samples (29.91%) were detected without pesticides.

Results of analysis of honeybees can be used to evaluate the exposure of pesticides for honeybees in every season. The number of samples per season (spring, summer and autumn) is shown in Fig. 3c. The detection of pesticide residues in individual honeybee samples at different seasons was shown in Fig. 3d. 77.78% of the honeybee samples tested in the spring contained pesticides, the highest proportion among the three seasons sampled. Notably, 1.85% of samples had more than 6 pesticides in the spring, while other seasons had no pesticides. With the peak period for controlling pests and diseases in spring, pesticide residue contamination in honeybee is the most serious.

Honeybees are exposed to a variety of pesticides throughout a variety of ways in an agricultural environment. The analysis revealed that 72.3% of the honeybee samples were contaminated by at least one compound in 2008 and 2009 in France^30^. Another work published that 73% of honeybee samples are positive to at least one plant protection product (PPP) from 2011 to 2013 in Greece^14^**.** Multiple residues prevailed in the honeybee samples, with 2 or more pesticides detected in 92.3% of the samples in North American^31^. Our experiment also has some limitations. It was neglected to detect acaricides residues of wintering bees (Fig. 3c)^32^. Other study detected the residue of Nis and its metabolites for honeybees, among the metabolites of imidacloprid were measured in both spring and summer samples, with a greater concentration in the spring samples than the summer, consistent with our finding (Fig. 3d)^33^. Besides all, the potential for cumulative toxicity of the exposure to several pesticides at the same time should also be considered(i.e. toxicity is more than additive)^34^. For instance, positive relationship between colony population disorders and detected fungicides was found^35^. The pyrethroids and ergosterol biosynthesis inhibitor (EBI) fungicides, propiconazole and thiamethoxam, the neonicotinoids and fungicides will generate synergism to increase toxicity to honeybees^36–38^.

### Toxicity of the four pyrethroid pesticides

After four pyrethroid pesticides exposure, the acute oral LD_50_ values of fenpropathrin and beta-cypermethrin for *A.m. ligustica* at 48 h were 0.2774, 0.1509 μμg/bee, which were 3.95 and 9.20 times higher than *A.c. cerana* were 0.0702, 0.0164 μg/bee, whereas lambda-cyhalothrin and bifenthrin had similar toxicity for *A.m. ligustica* and *A.c. cerana*, the toxicity of the first was 1.8 times higher than the second. These pesticides toxicity was beta-cypermethrin > fenpropathrin > lambda-cyhalothrin > bifenthrin. The results of this study suggested that the four pyrethroid pesticides were different toxic to these two honeybee species. The maximum determination concentrations of honeybee bodies for fenpropathrin, beta-cypermethrin, lambda-cyhalothrin, bifenthrin and the acute oral LD_50_ of two species of honeybees (Tables 1 and 2) were significantly different.

**Table 1:** The frequencies of detection and concentration of pesticide active ingredients in honeybee samples.

Pesticides	MQL (ng/g)	Positive sample	Detection rate (%)	Detected concentration (ng/g)		
				Mean	Median	Maximum
Carbendazim	2.5	74	34.6	160.2	15.8	2404.0
Fluvalinate	10.0	61	28.5	139.9	28.3	2802.3
Chlorpyrifos	5.0	41	19.2	33.9	9.2	838.4
Fenpropathrin	5.0	31	14.5	54.6	14.5	835.4
Pendimethalin	5	19	8.9	9.8	6.8	34.8
Diflubenzuron	5.0	18	8.4	16.9	13.4	59.8
Bifenthrin	2.5	10	4.7	71.0	63.9	172.8
Thiamethoxam	10.0	10	4.7	24.8	16.9	50.3
Pyridaben	5.0	10	4.7	37.9	15.5	220.4
Lambda-cyhalothrin	10.0	8	3.7	148.9	144.5	329.3
Trichlorfon	2.5	7	3.3	50.4	27.6	140.5
Pyrimethanil	2.5	7	3.3	13.2	5.6	50.8
Imidacloprid	10.0	5	2.3	30.5	31.0	68.8
Phoxim	5	5	2.3	7.9	9.0	9.2
Beta-cypermethrin	10.0	4	1.9	127.2	94.4	290.6
Coumaphos	5	3	1.4	278.4	109.0	671.8
Chlorothalonil	10.0	3	1.4	25.7	27.2	27.4
Azoxystrobin	2.5	3	1.4	4.6	5.0	5.5
Triadimefon	2.5	1	0.4	8.8	8.8	8.8
Omethoate	5.0	1	0.4	86.2	86.2	86.2
Acetamiprid	5.0	1	0.4	31.6	31.6	31.6

**Table 2:** Acute oral toxicity of four pyrethroids to *A.m. ligustica* and *A.c. cerana* at 48 h.

Pesticides	The species of bees	LD_50_ (µg/bee)	Fiducial limits (95%)	Linear regression equation	Linearity
Fenpropathrin	*A.m. ligustica*	0.2774	0.2214–0.3798	Y = 6.1999 + 2.1547x	0.9738
	*A.c. cerana*	0.0702	0.0494–0.0921	Y = 6.9722 + 1.7091x	0.9587
Beta-cypermethrin	*A.m. ligustica*	0.1509	0.1251–0.1841	Y = 6.8735 + 2.2813x	0.9623
	*A.c. cerana*	0.0164	0.0014–0.0318	Y = 8.8018 + 1.0089x	0.9453
Lambda-cyhalothrin	*A.m. ligustica*	0.2815	0.2395–0.3582	Y = 6.5741 + 2.8596x	0.9423
	*A.c. cerana*	0.1687	0.0852–0.2342	Y = 6.7377 + 2.2486x	0.9838
Bifenthrin	*A.m. ligustica*	0.3289	0.2914–0.3777	Y = 6.8122 + 3.7527x	0.9297
	*A.c. cerana*	0.1848	0.0795–0.2835	Y = 6.2215 + 1.6390x	0.9379

Two species of bees showed different sensitivity to the chemical pesticide. Our result showed that *A.c. cerana* is more sensitive than *A.m. ligustica* for beta-cypermethrin. Consistent with our findings, *A.m. ligustica* is more tolerant to malathion, cypermethrin, fenvalerate, deltamethrin and thiamethoxam than *A.c. cerana*^39^. However, *A.m. ligustica* and *A.c. cerana* showed opposite sensitivity to imidacloprid^40^. The main factor resulting in diverse LD_50_ values in two honeybee species remained uncertain. It may be due to the following factors, between biotic factors such as genetic distinction between different honeybee subspecies, colony differences in honeybees as well as physiology discrepancy of honeybees over different seasons, and abiotic factors, liking treatment temperature, the climatic condition and pesticide formulation ingredients^41–44^. In the majority of cases, the LD_50_ values of acute oral toxicity of two species of bees were much higher than the concentrations of pesticides detected in honeybee bodies. This may be related to rapid degradation and metabolism of pesticides in actual field conditions (temperature, light and humidity). Some real samples of beebread and honey did detect pesticide residues, while below known LD_50_ values for honeybees^45^. The detected concentrations values of tau-fluvalinate were lower than the median lethal dose (LD_50_) values^46^. But, some small quantities of active compounds can lead to chronic effects to various extents of honeybee physiology and behavior. However, it cannot be omitted that the cause of honeybee death incidents could have been related to these lower dose pesticides.

### Survival of the honeybees to beta-cypermethrin

**Figure 4:**
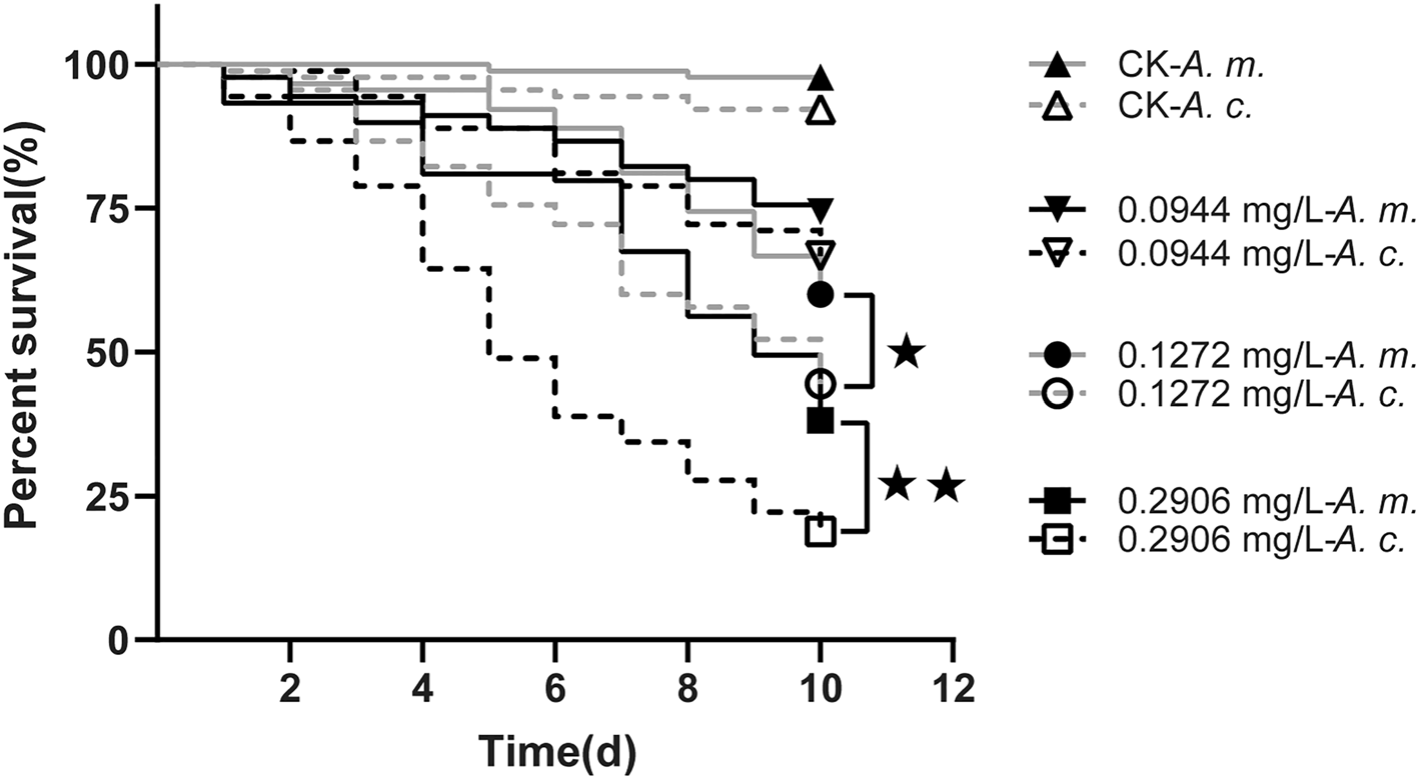
Survival risk analysis of beta-cypermethrin on *A. m. ligustica* and *A. c. cerana*. Based on our results for pesticide residue detection in honeybee nationwide, three concentrations were selected for beta-cypermethrin (Median, the median value of all samples; Mean, the mean value of all samples; Max, the maximum of all samples) as follow: 0.0944, 0.1272 and 0.2906 mg/L. Solvent treatment (acetone) served as blank control. Line with one star is significant different at *P* < 0.05 and two stars are significant different at *P* < 0.01.

The survival rates for *A. m. ligustica* and *A. c. cerana* after adults' consistent exposure (10 d) to pesticides were calculated to compare the chronic toxic effects. The survival rates for the forager bees of *A.m. ligustica* and *A.c. cerana* exposed to 0.1272, 0.2906 mg/L beta-cypermethrin were 60.0% and 44.4% (χ^2^ = 5.693, *p* = 0.017), 40.0% and 18.9% (χ^2^ = 17.573, *p* < 0.01), respectively (Fig. 4). There were no significant differences between survival rates of two species of bees fed a diet with any concentration of 0.0944 mg/L beta-cypermethrin and those fed solvent control diet (all *P* > 0.05). Two species of bees are different tolerance for chemicals*.* Our result showed that *A.c. cerana* is more sensitive than *A.m. ligustica* for beta-cypermethrin.

Preliminary pesticide risk assessment on honeybee is largely based on laboratory toxicity bioassays after both acute and chronic exposure. The effects and safety evaluation of pesticides on honeybees include acute and chronic toxicity studies at the laboratory level. Recent research reported that low-dose fenpropathrin significantly reduced the survival rate and homing ability of the workers, indicating that the pesticide has serious adverse effects on honeybee health^47^. Consistent with other studies, researcher found that some common pesticides and antibiotic exposure may have resulted in a decreased survival in the hive in previous studies^48–51^. Our work gives a glimpse of honeybees’ exposures to multiple pesticide residues. Hence, the pesticides used in the crops visited by honeybees during plant blooming should be severely restricted.

## Supplementary Information

Supplementary Information.

## Data Availability

The datasets used and/or analyzed during the current study are available from the corresponding author on reasonable request.
