# Supplementary material for: Toxic effects of detected pyrethroid pesticides on honeybee (Apis mellifera ligustica Spin and Apis cerana cerana Fabricius)
Source: Sci Rep. 2022 Oct 6;12:16695. doi: 10.1038/s41598-022-20925-x (PMC9537169; doi:10.1038/s41598-022-20925-x)
Supplement: Supplementary file 1 — Supplementary Information. [file 41598_2022_20925_MOESM1_ESM.docx]

**Supplementary Information**

**Toxic Effects of Detected Pyrethroid Pesticides on Honeybee (*Apis mellifera ligustica* Spin and *Apis cerana cerana* Fabricius)**

Qiongqiong Liu^1^, Qibao He^1^, Shiyu Zhang^1^, Yuhao Chai^1^, Quan Gao^1^, Jinjing Xiao^1^, Qingkui Fang^1^, Linsheng Yu^1^ & Haiqun Cao^1,2^*

^1^ *School of Plant Protection, Anhui Agricultural University, Hefei, 230036, China*

^2^ *Anhui Province Key Laboratory of Crop Integrated Pest Management, Hefei 230036, China*


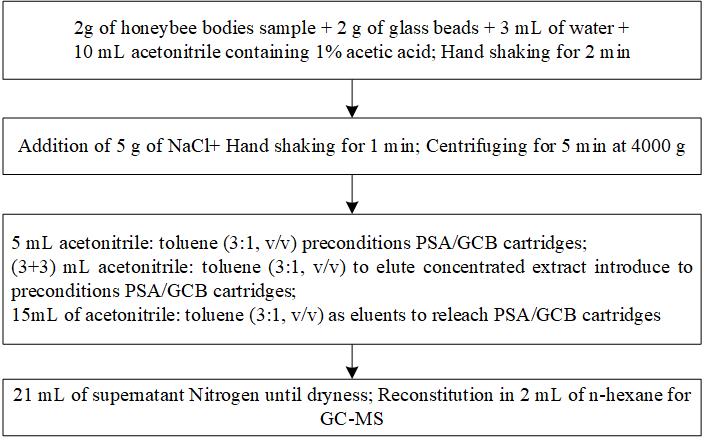


Figure S1. Diagram showing the process used to prepare honeybee samples for the GC-MS determination of pesticides

Table S1 Physicochemical properties, application dosage, and acceptable daily intake of pesticides and their main metabolites

| pesticides | CAS | Sw ^a^(mg·L^-1^) | Mode of action | ADI  (mg·kg^-1^ bw) ^c^ | Metabolites ^d^ |
| --- | --- | --- | --- | --- | --- |
| Methamidophos | 10265-92-6 | 2.0×10^5^ (20°C) | non-sys | 0.004 | ― |
| Acephate | 30560-19-1 | 7.9×10^5^ (20°C) | non-sys | 0.03 | Methamidophos |
| Omethoate | 1113-02-6 | 5.0×10^5^ (20°C) | sys ^b^ | 0.0003 | ― |
| Aldicarb-sulfoxide | 1646-87-3 | 2.8×10^4^ (20°C) | non-sys | ― | ― |
| Aldicarb-sulfone | 1646-88-4 | 1.0×10^4^ (25°C) | sys | ― | ― |
| Carbendazim | 10605-21-7 | 8.0 (20°C) | sys | 0.03 | 2-Aminobenzimidazole |
| Methomyl | 16752-77-5 | 5.5×10^4^ (20°C) | sys | 0.02 | Sulfamethomyl |
| Thiamethoxam | 153719-23-4 | 4.1×10^3^ (20 °C) | sys | 0.08 | clothianidin |
| Monocrotophos | 2157-98-4 | 8.2×10^5^ (20°C) | sys | 0.0006 | ― |
| Imidacloprid | 138261-41-3 | 610 (20°C) | sys | 0.06 | 6-chloro-pyridyl |
| Trichlorfon | 52-68-6 | 1.2×10^5^ (20°C) | non-sys | 0.002 | ― |
| Dimethoate | 60-51-5 | 2.5×10^4^ (20°C) | sys | 0.002 | Omethoate |
| Carbofuran-3-hydroxy | 16655-82-6 | 260.0 (25ºC) | non-sys | 0.001 | ― |
| Acetamiprid | 135410-20-7 | 2.9×10^3^ (20°C) | sys | 0.07 | ― |
| Aldicarb | 116-06-3 | 4.9×10^3^ (20°C) | sys | 0.003 | Aldicarb-sulfoxide、Aldicarb-sulfone |
| Phosphamidon | 13171-21-6 | 1.0×10^6^ (20°C) | non-sys | 0.0005 | ― |
| Dichlorvos | 62-73- | 1.8×10^4^ (20°C) | non-sys | 0.004 | ― |
| Carbofuran | 1563-66-2 | 322.0 (20°C) | sys | 0.001 | Carbofuran-3-hydroxy |
| Fenthion-sulfoxide | 3761-41-9 | 3.7 (20 ºC) | non-sys | ― | ― |
| Carbaryl | 63-25-2 | 9.1 (20°C) | non-sys | 0.008 | ― |
| Fenthion-sulfone | 3761-42-0 | ― | non-sys | ― | ― |
| Pyrimethanil | 53112-28-0 | 110.0 (20 ºC) | sys | 0.2 | ― |
| Phorate-sulfoxide | 2588-05-8 | 8.0×10^3^ (20°C) | non-sys | ― | ― |
| Phorate-sulfone | 2588-04-7 | 859.3 (19 ºC) | non-sys | ― | ― |
| Methidathion | 950-37-8 | 240.0 (20°C) | non-sys | 0.001 | ― |
| Phosmet | 732-11-6 | 15.2 (20°C) | non-sys | 0.01 | ― |
| Terbufos-sulfone | 56070-16-7 | 407.8 (18.5 ºC) | non-sys | ― | ― |
| Terbufos-sulfoxide | 10548-10-4 | >1×10^6^ (20°C) | non-sys | ― | ― |
| Azoxystrobin | 131860-33-8 | 6.7 (20°C) | sys | 0.2 | ― |
| Malathion | 121-75-5 | 148.0 (20°C) | non-sys | 0.3 | ― |
| Triadimefon | 43121-43-3 | 70.0 (20°C) | sys | 0.03 | Triadimenol |
| Dimethomorph | 110488-70-5 | 29.0 (20°C) | sys | 0.2 | Sum of isomers |
| Triazophos | 24017-47-8 | 35.0 (20°C) | non-sys | 0.001 | ― |
| Ethoprophos | 13194-48-4 | 1.3×10^3^ (20°C) | non-sys | 0.0004 | ― |
| Iprodione | 36734-19-7 | 6.8 (20°C) | non-sys | 0.06 | ― |
| Diflubenzuron | 35367-38-5 | 0.08 (20°C) | non-sys | 0.02 | ― |
| Procholraz | 67747-09-5 | 34.4 (25°C) | non-sys | ― | 2,4,6-trichlorophenol |
| Sulfotep | 3689-24-5 | 10.0 (20°C) | non-sys | 0.001 | ― |
| Chlorbenzuron | 35409-97-3 | 1.0 (20°C) | non-sys | 1.25 | ― |
| Fenthion | 55-38-9 | 4.2 (20°C) | non-sys | 0.007 | Fenthion-sulfone、Fenthion-sulfoxide |
| Coumaphos | 56-72-4 | 1.5 (20°C) | non-sys | 0.0003 | ― |
| Diazinon | 333-41-5 | 60.0 (20°C) | non-sys | 0.005 | ― |
| Phoxim | 14816-18-3 | 1.5 (20 °C) | non-sys | 0.004 | ― |
| Phorate | 298-02-2 | 50.0 (20°C) | sys | 0.0007 | Phorate-sulfoxid、Phorate-sulfone |
| Phosalone | 2310-17-0 | 1.4 (20°C) | non-sys | 0.02 | ― |
| Difenoconazole | 119446-68-3 | 15.0 (20ºC) | sys | 0.01 | ― |
| Emamectin benzoate | 155569-91-8 | Slightly soluble | non-sys | 0.0005 | Emamectin B1a |
| Profenofos | 41198-08-7 | 28.0 (20°C) | non-sys | 0.03 | ― |
| Terbufos | 13071-79-9 | 4.5 (20℃) | sys | 0.0006 | Terbufos-sulfone、  Terbufos-sulfoxide |
| Chlorpyrifos | 2921-88-2 | 1.1 (20℃) | non-sys | 0.01 | ― |
| Fenpropathrin | 64257-84-7 | 0.33 (20℃) | non-sys | 0.03 | ― |
| Pendimethalin | 40487-42-1 | 0.33 (20°C) | non-sys | 0.1 | ― |
| Pyridaben | 96489-71-3 | 0.022 (20°C) | non-sys | 0.01 | ― |
| Fluvalinate | 69409-94-5 | 0.002 (20℃) | non-sys | 0.005 | ― |
| Chlorothalonil | 1897-45-6 | 0.81 (20°C) | non-sys | 0.02 | 4-hydroxy chlorothalonil |
| Alachlor | 15972-60-8 | 240.0 (20°C) | non-sys | 0.01 | ― |
| Metolachlor | 51218-45-2 | 530.0 (20℃) | non-sys | 0.1 | ― |
| Butralin | 33629-47-9 | 0.308 (20°C) | non-sys | 0.2 | ― |
| Oxadiazon | 19666-30-9 | 0.57 (20ºC) | non-sys | 0.0036 | ― |
| Bifenthrin | 82657-04-3 | 0.001 (20°C) | non-sys | 0.01 | Sum of isomers |
| Lambda-cyhalothrin | 91465-08-6 | 0.005 (20℃) | non-sys | 0.02 | Sum of isomers |
| Beta-cypermethrin | 52315-07-8 | 0.9 (20℃） | non-sys | 0.02 | Sum of isomers |
| Boscalid | 188425-85-6 | 4.6 (20℃） | non-sys | 0.04 | ― |
| Quizalofop-p-ethyl | 100646-51-3 | 0.61 (20°C) | non-sys | 0.0009 | Quizalofop-p-ethyl acid |
| Fenvalerate | 51630-58-1 | 0.001 (20℃） | non-sys | 0.02 | Sum of isomers |
| Deltamethrin | 52820-00-5 | 0.002 (20℃） | non-sys | 0.01 | Sum of isomers |

^a^ Sw, solubility in water. ^b^ sys, systemic pesticide. ^c^ ADI, obtained from the GB 2763-2019, China (National Food Safety Standard-maximum residue limits for pesticides in food). ^d^ Metabolites, obtained from Pesticide registration residue test residues to be tested and dietary risk assessment residue definition catalog.

Table S2 Ion transitions used for the quantification and confirmation and dwell time in GC-MS

| Pesticide | Quantification | Confirmation 1 | Confirmation 2 | dwell time (ms) |
| --- | --- | --- | --- | --- |
| Chlorothalonil | 264 | 266 | 269 | 80 |
| Alachlor | 45 | 160 | 188 | 120 |
| Metolachlor | 162 | 238 | 45 | 100 |
| Butralin | 266 | 224 | 220 | 60 |
| Oxadiazon | 177 | 41 | 176 | 120 |
| Bifenthrin | 181 | 165 | 166 | 60 |
| Lambda-cyhalothrin | 181 | 197 | 208 | 60 |
| Beta-cypermethrin | 163 | 181 | 165 | 60 |
| Boscalid | 140 | 112 | 142 | 60 |
| Quizalofop-p-ethyl | 299 | 372 | 163 | 60 |
| Fenvalerate | 125 | 167 | 225 | 60 |
| Deltamethrin | 181 | 253 | 77 | 60 |

Table S3 Ion transitions used for the quantification (MRM1) and confirmation (MRM2), dwell time, cone voltage and collision energy UPLC-MS/MS settings

| **Pesticide** | **Transitions** | **Dwell Time (ms)** | **Cone Voltage (V)** | **Collision Energy (eV)** |
| --- | --- | --- | --- | --- |
| Methamidophos | Quantification ion142 >93.9 | 50 | 17 | 13 |
|  | Confirmation ion142 >124.9 |  | 17 | 13 |
| Acephate | Quantification ion184.1 >143 | 36 | 8 | 8 |
|  | Confirmation ion184.1 >125.1 |  | 8 | 18 |
| Omethoate | Quantification ion214.1 >125.1 | 28 | 16 | 22 |
|  | Confirmation ion214.1 >183.1 |  | 16 | 11 |
| Aldicarb sulfoxide | Quantification ion207 >89 | 28 | 13 | 14 |
|  | Confirmation ion207 >132 |  | 13 | 10 |
| Aldicarb sulfone | Quantification ion223 >86 | 28 | 22 | 14 |
|  | Confirmation ion223 >148 |  | 22 | 10 |
| Carbendazim | Quantification ion192.1 >160.1 | 28 | 24 | 18 |
|  | Confirmation ion192.1 >132.1 |  | 24 | 28 |
| Methomyl | Quantification ion163 >88 | 28 | 17 | 10 |
|  | Confirmation ion163 >106 |  | 17 | 10 |
| Thiamethoxam | Quantification ion292.1 >210.9 | 44 | 18 | 12 |
|  | Confirmation ion292.1 >181 |  | 18 | 24 |
| Monocrotophos | Quantification ion224.1 >127.1 | 44 | 15 | 16 |
|  | Confirmation ion224.1 >98.1 |  | 15 | 12 |
| Imidacloprid | Quantification ion256.1 >209.1 | 28 | 23 | 15 |
|  | Confirmation ion256.1 >175.1 |  | 23 | 20 |
| Trichlorfon | Quantification ion257 >109 | 28 | 22 | 18 |
|  | Confirmation ion257 >79 |  | 22 | 30 |
| Dimethoate | Quantification ion230.1 >199 | 28 | 12 | 10 |
|  | Confirmation ion230.1 >125 |  | 12 | 20 |
| Carbofuran-3-hydroxy | Quantification ion238 >163 | 28 | 25 | 16 |
|  | Confirmation ion238 >181 |  | 25 | 10 |
| Acetamiprid | Quantification ion223 >126 | 28 | 23 | 20 |
|  | Confirmation ion223 >56.1 |  | 23 | 15 |
| Aldicarb | Quantification ion212.8 >88.9 | 78 | 20 | 16 |
|  | Confirmation ion212.8 >115.9 |  | 20 | 12 |
| Phosphamidon | Quantification ion300.1 >174.1 | 28 | 17 | 14 |
|  | Confirmation ion300.1 >127.1 |  | 17 | 25 |
| Dichlorvos | Quantification ion221 >109 | 22 | 23 | 22 |
|  | Confirmation ion221 >79 |  | 23 | 34 |
| Carbofuran | Quantification ion222.1 >165.1 | 22 | 25 | 16 |
|  | Confirmation ion222.1 >123 |  | 25 | 16 |
| Fenthion-sulfoxide | Quantification ion295 >109 | 22 | 29 | 32 |
|  | Confirmation ion295 >280 |  | 29 | 18 |
| Carbaryl | Quantification ion202 >145 | 22 | 19 | 22 |
|  | Confirmation ion202 >117 |  | 19 | 28 |
| Fenthion-sulfone | Quantification ion311 >125 | 22 | 29 | 22 |
|  | Confirmation ion311 >109 |  | 29 | 28 |
| Pyrimethanil | Quantification ion200.2 >107 | 22 | 42 | 24 |
|  | Confirmation ion200.2 >82 |  | 42 | 24 |
| Phorate-sulfoxide | Quantification ion277 >96.9 | 22 | 15 | 32 |
|  | Confirmation ion277 >143 |  | 15 | 20 |
| Phorate-sulfone | Quantification ion293 >96.9 | 22 | 15 | 30 |
|  | Confirmation ion293 >115 |  | 15 | 24 |
| Methidathion | Quantification ion303 >145 | 22 | 10 | 10 |
|  | Confirmation ion303 >85.1 |  | 10 | 20 |
| Phosmet | Quantification ion318 >160 | 18 | 20 | 14 |
|  | Confirmation ion340 >214.1 |  | 30 | 14 |
| Terbufos-sulfone | Quantification ion321.2 >171 | 18 | 19 | 12 |
|  | Confirmation ion321.2 >97 |  | 19 | 40 |
| Terbufos-sulfoxide | Quantification ion305 >187 | 18 | 10 | 11 |
|  | Confirmation ion305 >97 |  | 10 | 40 |
| Azoxystrobin | Quantification ion404 >372 | 17 | 17 | 15 |
|  | Confirmation ion404 >329 |  | 17 | 30 |
| Malathion | Quantification ion331 >127 | 18 | 18 | 12 |
|  | Confirmation ion331 >79 |  | 18 | 40 |
| Triadimefon | Quantification ion294.1 >197.2 | 18 | 22 | 15 |
|  | Confirmation ion294.1 >69.3 |  | 22 | 20 |
| Dimethomorph | Quantification ion388.1 >300.9 | 18 | 30 | 20 |
|  | Confirmation ion388.1 >165 |  | 30 | 30 |
| Triazophos | Quantification ion314.1 >161.9 | 13 | 22 | 18 |
|  | Confirmation ion314.1 >118.9 |  | 22 | 35 |
| Ethoprophos | Quantification ion243.2 >131 | 8 | 18 | 20 |
|  | Confirmation ion243.2 >97 |  | 18 | 31 |
| Iprodione | Quantification ion330 >244.7 | 8 | 12 | 16 |
|  | Confirmation ion330 >288 |  | 12 | 15 |
| Diflubenzuron | Quantification ion310.9 >157.9 | 8 | 20 | 14 |
|  | Confirmation ion310.9 >140.9 |  | 20 | 36 |
| Procholraz | Quantification ion376 >308 | 8 | 20 | 15 |
|  | Confirmation ion376 >266 |  | 20 | 15 |
| Sulfotep | Quantification ion323 >97 | 8 | 17 | 32 |
|  | Confirmation ion323 >171 |  | 17 | 15 |
| Chlorbenzuron | Quantification ion309 >155.9 | 8 | 22 | 26 |
|  | Confirmation ion309 >138.8 |  | 22 | 18 |
| Fenthion | Quantification ion279 >168.9 | 8 | 30 | 18 |
|  | Confirmation ion279 >105 |  | 30 | 28 |
| Coumaphos | Quantification ion363.1 >307 | 8 | 21 | 16 |
|  | Confirmation ion363.1 >289 |  | 21 | 24 |
| Diazinon | Quantification ion305.1 >169 | 8 | 20 | 22 |
|  | Confirmation ion305.1 >96.9 |  | 20 | 35 |
| Phoxim | Quantification ion299 >129 | 8 | 12 | 13 |
|  | Confirmation ion299 >153 |  | 12 | 7 |
| Phorate | Quantification ion261 >97 | 8 | 14 | 28 |
|  | Confirmation ion261 >75 |  | 14 | 10 |
| Phosalone | Quantification ion367.9 >181.9 | 8 | 12 | 14 |
|  | Confirmation ion367.9 >110.9 |  | 12 | 42 |
| Difenoconazole | Quantification ion406 >251.1 | 26 | 37 | 25 |
|  | Confirmation ion406 >111.1 |  | 37 | 60 |
| Emamectin benzoate | Quantification ion886.5 >158.1 | 22 | 20 | 32 |
|  | Confirmation ion886.5 >81.9 |  | 20 | 64 |
| Profenofos | Quantification ion372.9 >302.6 | 22 | 25 | 20 |
|  | Confirmation ion372.9 >127.9 |  | 25 | 40 |
| Terbufos | Quantification ion289 >103 | 22 | 12 | 8 |
|  | Confirmation ion289 >57.2 |  | 12 | 22 |
| Chlorpyrifos | Quantification ion350 >97 | 22 | 27 | 32 |
|  | Confirmation ion350 >198 |  | 27 | 20 |
| Fenpropathrin | Quantification ion350.1 >97 | 22 | 15 | 34 |
|  | Confirmation ion350.1 >125 |  | 15 | 14 |
| Pendimethalin | Quantification ion252.2 >212.2 | 22 | 12 | 10 |
|  | Confirmation ion252.2 >194.1 |  | 12 | 17 |
| Pyridaben | Quantification ion365.1 >147.1 | 22 | 19 | 24 |
|  | Confirmation ion365.1 >309.1 |  | 19 | 12 |
| Fluvalinate | Quantification ion507 >181.1 | 22 | 15 | 30 |
|  | Confirmation ion507 >208.1 |  | 15 | 12 |

Table S4 Method limit of determination and quantification (LOD and LOQ), linear range, linear regression equation and linearity

| **Pesticide** | **LOD (ng/g)** | **LOQ (ng/g)** | **LinearRane (ng/g)** | **Linear Regression Equation** | **Linearity** |
| --- | --- | --- | --- | --- | --- |
| Methamidophos | 0.0556 | 0.1667 | 5–200 | Y = 140.5X + 99.14 | 0.9984 |
| Acephate | 0.2691 | 0.8072 | 5–200 | Y = 61.56X − 69.02 | 0.9990 |
| Omethoate | 0.1383 | 0.4149 | 5–200 | Y = 266.0X + 94.55 | 0.9963 |
| Aldicarb-sulfoxide | 0.5291 | 1.5873 | 5–200 | Y = 38.60X + 39.18 | 0.9954 |
| Aldicarb-sulfone | 0.1343 | 0.4030 | 5–200 | Y = 112.0X + 38.23 | 0.9994 |
| Carbendazim | 0.1064 | 0.3191 | 2.5–100 | Y = 941.6X + 11.03 | 0.9993 |
| Methomyl | 0.0337 | 0.1010 | 5–200 | Y = 98.08X − 28.38 | 0.9997 |
| Thiamethoxam | 0.0028 | 0.0084 | 10–400 | Y = 66.00X + 134.2 | 0.9975 |
| Monocrotophos | 0.0051 | 0.0154 | 5–200 | Y = 1022X + 889.6 | 0.9967 |
| Imidacloprid | 0.0809 | 0.2427 | 10–400 | Y = 71.91X − 48.57 | 0.9996 |
| Trichlorfon | 0.1265 | 0.3794 | 5–200 | Y = 138.7X + 12.85 | 0.9991 |
| Dimethoate | 0.0366 | 0.1098 | 5–200 | Y = 154.1X + 79.95 | 0.9986 |
| Carbofuran-3-hydroxy | 0.0344 | 0.1032 | 5–200 | Y = 179.0X + 101.9 | 0.9991 |
| Acetamiprid | 0.0114 | 0.0343 | 5–200 | Y = 645.1X + 279.1 | 0.9986 |
| Aldicarb | 0.0432 | 0.1295 | 5–200 | Y = 578.5X + 620.2 | 0.9954 |
| Phosphamidon | 0.0037 | 0.0112 | 2.5–100 | Y = 207.4X − 73.14 | 0.9996 |
| Dichlorvos | 0.2483 | 0.7450 | 5–200 | Y = 232.7X − 24.55 | 0.9998 |
| Carbofuran | 0.0060 | 0.0179 | 5–200 | Y = 854.1X − 164.8 | 0.9995 |
| Fenthion-sulfoxide | 0.0202 | 0.0605 | 5–200 | Y = 780.0X + 66.08 | 0.9999 |
| Carbaryl | 0.1087 | 0.3261 | 5–200 | Y = 139.9X + 21.77 | 0.9985 |
| Fenthion-sulfone | 0.0369 | 0.1108 | 5–200 | Y = 165.1X − 26.61 | 0.9991 |
| Pyrimethanil | 0.0145 | 0.0435 | 2.5–100 | Y = 1810X − 63.22 | 0.9999 |
| Phorate-sulfoxide | 0.0244 | 0.0731 | 5–200 | Y = 938.3X + 576.2 | 0.9990 |
| Phorate-sulfone | 0.0241 | 0.0722 | 5–200 | Y = 302.9X − 5.827 | 0.9998 |
| Methidathion | 0.0095 | 0.0286 | 5–200 | Y = 93.08X + 9.293 | 0.9998 |
| Phosmet | 0.0343 | 0.1029 | 5–200 | Y = 106.4X + 64.63 | 0.9976 |
| Terbufos-sulfone | 0.0759 | 0.2277 | 5–200 | Y = 96.91X − 66.94 | 0.9975 |
| Terbufos-sulfoxide | 0.0243 | 0.0729 | 5–200 | Y = 192.5X − 80.41 | 0.9983 |
| Azoxystrobin | 0.0062 | 0.0186 | 2.5–100 | Y = 363.1X + 48.65 | 0.9990 |
| Malathion | 0.0610 | 0.1829 | 5–200 | Y = 151.5X − 111.6 | 0.9952 |
| Triadimefon | 0.0029 | 0.0088 | 2.5–100 | Y = 310.0X − 135.0 | 0.9955 |
| Dimethomorph | 0.0049 | 0.0147 | 5–200 | Y = 168.5X − 169.4 | 0.9903 |
| Triazophos | 0.0082 | 0.0246 | 2.5–100 | Y = 747.9X + 45.03 | 0.9995 |
| Ethoprophos | 0.0177 | 0.0530 | 5–200 | Y = 305.2X − 87.03 | 0.9992 |
| Iprodione | 0.0611 | 0.1833 | 10–400 | Y = 55.78X − 106.2 | 0.9952 |
| Diflubenzuron | 0.0045 | 0.0136 | 5–200 | Y = 86.59X − 52.92 | 0.9941 |
| Procholraz | 0.0166 | 0.0499 | 5–200 | Y = 587.4X − 606.3 | 0.9902 |
| Sulfotep | 0.0195 | 0.0585 | 5–200 | Y = 1144X − 1042 | 0.9955 |
| Chlorbenzuron | 0.0226 | 0.0678 | 5–200 | Y = 89.28X − 107.6 | 0.9940 |
| Fenthion | 0.0514 | 0.1542 | 5–200 | Y = 101.6X − 19.60 | 0.9983 |
| Coumaphos | 0.0030 | 0.0090 | 5–200 | Y = 63.55X − 95.86 | 0.9914 |
| Diazinon | 0.0176 | 0.0529 | 2.5–100 | Y = 1220X − 596.6 | 0.9907 |
| Phoxim | 0.0135 | 0.0406 | 5–200 | Y = 49.86X + 5.412 | 0.9969 |
| Phorate | 0.0154 | 0.0462 | 5–200 | Y = 33.04X − 65.92 | 0.9996 |
| Phosalone | 0.0158 | 0.0475 | 5–200 | Y = 49.75X − 34.82 | 0.9944 |
| Difenoconazole | 0.0242 | 0.0726 | 5–200 | Y = 520.7X − 417.0 | 0.9949 |
| Emamectin benzoate | 0.0008 | 0.0025 | 5–200 | Y = 1177X − 804.8 | 0.9931 |
| Profenofos | 0.0189 | 0.0568 | 5–200 | Y = 182.7X − 171.3 | 0.9935 |
| Terbufos | 0.1414 | 0.4241 | 5–200 | Y = 83.82X − 1.636 | 0.9989 |
| Chlorpyrifos | 0.0638 | 0.1914 | 5–200 | Y = 327.6X + 67.46 | 0.9995 |
| Fenpropathrin | 0.0433 | 0.1300 | 5–200 | Y = 407.6X + 67.03 | 0.9999 |
| Pendimethalin | 0.0236 | 0.0708 | 5–200 | Y = 230.1X − 83.93 | 0.9987 |
| Pyridaben | 0.0070 | 0.0211 | 5–200 | Y = 1811X − 172.8 | 0.9984 |
| Fluvalinate | 0.0068 | 0.0203 | 10–400 | Y = 472.3X + 334.3 | 0.9997 |
| Chlorothalonil | 0.105 | 0.316 | 5-200 | Y=207462X-226 | 0.9980 |
| Alachlor | 2.222 | 6.667 | 10-400 | Y=41907X-1125 | 0.9974 |
| Metolachlor | 0.028 | 0.083 | 2.5-100 | Y=325114X-10 | 0.9989 |
| Butralin | 0.238 | 0.714 | 5-200 | Y=68744X-241 | 0.9985 |
| Oxadiazon | 0.309 | 0.926 | 5-200 | Y=85744X-162 | 0.9997 |
| Bifenthrin | 0.025 | 0.075 | 2.5-100 | Y=306445X-250 | 0.9993 |
| Lambda-cyhalothrin | 0.214 | 0.641 | 10-400 | Y=72254X-968 | 0.9976 |
| Beta-cypermethrin | 2.381 | 7.143 | 10-400 | Y=47614X-726 | 0.9961 |
| Boscalid | 0.833 | 2.500 | 10-400 | Y=77782X-1052 | 0.9979 |
| Quizalofop-p-ethyl | 2.436 | 7.308 | 10-400 | Y=14910X-58 | 0.9990 |
| Fenvalerate | 1.389 | 4.167 | 10-400 | Y=42834X-302 | 0.9997 |
| Deltamethrin | 2.586 | 7.758 | 10-400 | Y=15517X+33 | 0.9991 |

Table S5 Recoveries, RSDs (relative standard deviations) and MEs (matrix effects) of 66 pesticides in Honeybee matrices

| Pesticide No. | Pesticide | Spiking level (ng/g) | Recovery (%) | RSD (%) | ME (%) |
| --- | --- | --- | --- | --- | --- |
| 1 | Methamidophos | 5 | 99.33 | 2.32 | -9 |
|  |  | 50 | 102.80 | 4.68 |  |
|  |  | 500 | 105.67 | 5.63 |  |
| 2 | Acephate | 5 | 98.00 | 9.35 | 1 |
|  |  | 50 | 96.80 | 2.38 |  |
|  |  | 500 | 112.67 | 6.54 |  |
| 3 | Omethoate | 5 | 104.00 | 1.92 | -18 |
|  |  | 50 | 106.53 | 0.76 |  |
|  |  | 500 | 113.73 | 5.28 |  |
| 4 | Aldicarb-sulfoxide | 5 | 99.33 | 2.32 | -2 |
|  |  | 50 | 106.13 | 3.06 |  |
|  |  | 500 | 116.67 | 5.55 |  |
| 5 | Aldicarb-sulfone | 5 | 127.33 | 7.25 | -10 |
|  |  | 50 | 105.60 | 4.64 |  |
|  |  | 500 | 116.27 | 5.74 |  |
| 6 | Carbendazim | 2.5 | 104.00 | 3.85 | 8 |
|  |  | 25 | 102.67 | 0.90 |  |
|  |  | 250 | 115.60 | 6.35 |  |
| 7 | Methomyl | 5 | 68.00 | 2.94 | 5 |
|  |  | 50 | 70.93 | 8.10 |  |
|  |  | 500 | 99.07 | 5.14 |  |
| 8 | Thiamethoxam | 10 | 107.67 | 2.68 | -20 |
|  |  | 100 | 117.70 | 4.56 |  |
|  |  | 1000 | 117.33 | 6.91 |  |
| 9 | Monocrotophos | 5 | 108.67 | 1.06 | 15 |
|  |  | 50 | 109.20 | 1.94 |  |
|  |  | 500 | 117.13 | 5.17 |  |
| 10 | Imidacloprid | 10 | 127.67 | 0.45 | -13 |
|  |  | 100 | 116.17 | 15.66 |  |
|  |  | 1000 | 120.27 | 7.59 |  |
| 11 | Trichlorfon | 5 | 72.67 | 8.41 | -15 |
|  |  | 50 | 93.53 | 7.29 |  |
|  |  | 500 | 111.33 | 5.81 |  |
| 12 | Dimethoate | 5 | 115.33 | 3.61 | -20 |
|  |  | 50 | 112.13 | 3.27 |  |
|  |  | 500 | 116.47 | 7.30 |  |
| 13 | Carbofuran-3-hydroxy | 5 | 113.33 | 3.67 | -40 |
|  |  | 50 | 115.53 | 6.52 |  |
|  |  | 500 | 117.60 | 7.51 |  |
| 14 | Acetamiprid | 5 | 120.67 | 1.91 | 30 |
|  |  | 50 | 113.07 | 2.58 |  |
|  |  | 500 | 117.93 | 5.40 |  |
| 15 | Aldicarb | 5 | 108.67 | 2.13 | -24 |
|  |  | 50 | 111.47 | 10.26 |  |
|  |  | 500 | 117.67 | 6.41 |  |
| 16 | Phosphamidon | 2.5 | 113.33 | 2.04 | 15 |
|  |  | 25 | 110.40 | 1.66 |  |
|  |  | 250 | 116.40 | 5.13 |  |
| 17 | Dichlorvos | 5 | 104.00 | 1.92 | -14 |
|  |  | 50 | 92.67 | 3.59 |  |
|  |  | 500 | 100.13 | 0.61 |  |
| 18 | Carbofuran | 5 | 102.00 | 3.40 | -38 |
|  |  | 50 | 110.67 | 1.72 |  |
|  |  | 500 | 115.07 | 6.83 |  |
| 19 | Fenthion-sulfoxide | 5 | 117.33 | 1.97 | -51 |
|  |  | 50 | 113.40 | 2.17 |  |
|  |  | 500 | 120.87 | 6.64 |  |
| 20 | Carbaryl | 5 | 88.00 | 6.82 | -30 |
|  |  | 50 | 75.47 | 7.36 |  |
|  |  | 500 | 93.00 | 7.25 |  |
| 21 | Fenthion-sulfone | 5 | 114.00 | 3.04 | -44 |
|  |  | 50 | 109.67 | 5.84 |  |
|  |  | 500 | 125.47 | 6.97 |  |
| 22 | Pyrimethanil | 2.5 | 97.33 | 2.37 | -23 |
|  |  | 25 | 104.00 | 3.71 |  |
|  |  | 250 | 112.00 | 9.24 |  |
| 23 | Phorate-sulfoxide | 5 | 110.67 | 5.22 | 27 |
|  |  | 50 | 117.47 | 3.58 |  |
|  |  | 500 | 121.20 | 7.31 |  |
| 24 | Phorate-sulfone | 5 | 124.00 | 5.59 | -24 |
|  |  | 50 | 112.40 | 1.75 |  |
|  |  | 500 | 122.47 | 7.60 |  |
| 25 | Methidathion | 5 | 112.00 | 1.79 | -4 |
|  |  | 50 | 116.40 | 0.91 |  |
|  |  | 500 | 123.13 | 8.13 |  |
| 26 | Phosmet | 5 | 111.33 | 2.07 | 50 |
|  |  | 50 | 107.60 | 9.05 |  |
|  |  | 500 | 120.67 | 9.25 |  |
| 27 | Terbufos-sulfone | 5 | 118.67 | 3.89 | 16 |
|  |  | 50 | 117.33 | 3.77 |  |
|  |  | 500 | 117.20 | 6.45 |  |
| 28 | Terbufos-sulfoxide | 5 | 118.00 | 1.69 | 22 |
|  |  | 50 | 115.67 | 3.84 |  |
|  |  | 500 | 119.07 | 8.23 |  |
| 29 | Azoxystrobin | 2.5 | 109.33 | 7.62 | 13 |
|  |  | 25 | 120.80 | 2.17 |  |
|  |  | 250 | 119.60 | 6.68 |  |
| 30 | Malathion | 5 | 116.00 | 1.72 | 25 |
|  |  | 50 | 116.33 | 4.39 |  |
|  |  | 500 | 117.60 | 6.35 |  |
| 31 | Triadimefon | 2.5 | 120.00 | 6.67 | 15 |
|  |  | 25 | 110.27 | 4.13 |  |
|  |  | 250 | 117.60 | 7.66 |  |
| 32 | Dimethomorph | 5 | 104.67 | 3.98 | -20 |
|  |  | 50 | 112.60 | 4.78 |  |
|  |  | 500 | 102.20 | 6.45 |  |
| 33 | Triazophos | 2.5 | 108.00 | 3.70 | 16 |
|  |  | 25 | 113.73 | 1.94 |  |
|  |  | 250 | 115.60 | 7.32 |  |
| 34 | Ethoprophos | 5 | 98.00 | 6.12 | -1 |
|  |  | 50 | 106.67 | 1.52 |  |
|  |  | 500 | 114.20 | 4.47 |  |
| 35 |  | 10 | 89.00 | 4.49 |  |
|  | Iprodione | 100 | 113.67 | 8.44 | 16 |
|  |  | 1000 | 111.53 | 7.16 |  |
| 36 | Diflubenzuron | 5 | 112.67 | 2.05 | 8 |
|  |  | 50 | 113.60 | 4.91 |  |
|  |  | 500 | 101.33 | 5.98 |  |
| 37 | Procholraz | 5 | 113.33 | 2.70 | -27 |
|  |  | 50 | 105.40 | 3.64 |  |
|  |  | 500 | 101.87 | 7.91 |  |
| 38 | Sulfotep | 5 | 108.00 | 4.90 | -33 |
|  |  | 50 | 105.73 | 5.24 |  |
|  |  | 500 | 111.67 | 3.85 |  |
| 39 | Chlorbenzuron | 5 | 112.67 | 8.39 | 4 |
|  |  | 50 | 111.13 | 9.79 |  |
|  |  | 500 | 102.87 | 10.03 |  |
| 40 | Fenthion | 5 | 99.33 | 9.30 | -66 |
|  |  | 50 | 116.33 | 5.76 |  |
|  |  | 500 | 113.73 | 8.03 |  |
| 41 | Coumaphos | 5 | 95.33 | 5.28 | -35 |
|  |  | 50 | 120.93 | 9.44 |  |
|  |  | 500 | 115.73 | 4.96 |  |
| 42 | Diazinon | 2.5 | 106.67 | 2.17 | 22 |
|  |  | 25 | 108.00 | 2.59 |  |
|  |  | 250 | 111.73 | 4.45 |  |
| 43 | Phoxim | 10 | 118.00 | 16.69 | -20 |
|  |  | 100 | 110.4 | 4.62 |  |
|  |  | 1000 | 123.07 | 10.11 |  |
| 44 | Phorate | 5 | 107.33 | 15.06 | 9 |
|  |  | 50 | 125.07 | 6.72 |  |
|  |  | 500 | 120.13 | 5.83 |  |
| 45 | Phosalone | 5 | 100.67 | 8.03 | 8 |
|  |  | 50 | 117.93 | 5.34 |  |
|  |  | 500 | 107.80 | 5.48 |  |
| 46 | Difenoconazole | 5 | 86.00 | 4.65 | 14 |
|  |  | 50 | 108.07 | 5.18 |  |
|  |  | 500 | 100.87 | 2.66 |  |
| 47 | Emamectin benzoate | 5 | 94.00 | 4.26 | 4 |
|  |  | 50 | 98.93 | 9.55 |  |
|  |  | 500 | 86.73 | 1.16 |  |
| 48 | Profenofos | 5 | 101.33 | 2.28 | 1 |
|  |  | 50 | 105.33 | 7.13 |  |
|  |  | 500 | 105.47 | 7.66 |  |
| 49 | Terbufos | 5 | 94.00 | 4.26 | -9 |
|  |  | 50 | 100.67 | 7.77 |  |
|  |  | 500 | 110.33 | 6.93 |  |
| 50 | Chlorpyrifos | 5 | 92.67 | 6.59 | -17 |
|  |  | 50 | 101.53 | 6.67 |  |
|  |  | 500 | 109.73 | 6.46 |  |
| 51 | Fenpropathrin | 5 | 100.00 | 7.21 | -24 |
|  |  | 50 | 105.67 | 8.64 |  |
|  |  | 500 | 107.53 | 3.81 |  |
| 52 | Pendimethalin | 5 | 100.00 | 9.17 | 1 |
|  |  | 50 | 104.40 | 5.21 |  |
|  |  | 500 | 109.07 | 5.42 |  |
| 53 | Pyridaben | 2.5 | 98.67 | 2.34 | 27 |
|  |  | 25 | 98.67 | 4.48 |  |
|  |  | 250 | 104.40 | 5.07 |  |
| 54 | Fluvalinate | 10 | 74.67 | 6.74 | -50 |
|  |  | 100 | 90.77 | 3.25 |  |
|  |  | 1000 | 102.90 | 5.20 |  |
| 55 | Chlorothalonil | 10 | 83 | 8 | 49 |
|  |  | 100 | 76 | 14 |  |
|  |  | 1000 | 75 | 3 |  |
| 56 | Alachlor | 5 | 97 | 7 | 28 |
|  |  | 50 | 89 | 7 |  |
|  |  | 500 | 99 | 4 |  |
| 57 | Melachlor | 2.5 | 81 | 4 | 17 |
|  |  | 25 | 98 | 3 |  |
|  |  | 250 | 108 | 5 |  |
| 58 | Butralin | 5 | 104 | 9 | 11 |
|  |  | 50 | 88 | 2 |  |
|  |  | 500 | 89 | 6 |  |
| 59 | Oxadiazon | 5 | 95 | 4 | 32 |
|  |  | 50 | 98 | 5 |  |
|  |  | 500 | 107 | 2 |  |
| 60 | Bifenthrin | 2.5 | 88 | 5 | 79 |
|  |  | 25 | 90 | 2 |  |
|  |  | 250 | 105 | 7 |  |
| 61 | Lambda-cyhalothrin | 10 | 103 | 3 | 89 |
|  |  | 100 | 99 | 4 |  |
|  |  | 1000 | 112 | 5 |  |
| 62 | Beta-cypermethrin | 10 | 101 | 5 | 19 |
|  |  | 100 | 97 | 7 |  |
|  |  | 1000 | 93 | 8 |  |
| 63 | Boscalid | 10 | 74 | 8 | 98 |
|  |  | 100 | 81 | 13 |  |
|  |  | 1000 | 86 | 11 |  |
| 64 | Quizalofop-p-ethyl | 10 | 77 | 4 | 35 |
|  |  | 100 | 99 | 5 |  |
|  |  | 1000 | 93 | 6 |  |
| 65 | Fenvalerate | 10 | 84 | 18 | 29 |
|  |  | 100 | 97 | 6 |  |
|  |  | 1000 | 101 | 4 |  |
| 66 | Deltamethrin | 10 | 96 | 10 | 47 |
|  |  | 100 | 100 | 5 |  |
|  |  | 1000 | 86 | 7 |  |
